# Early recognition of acute thoracic aortic dissection and aneurysm

**DOI:** 10.1186/1749-7922-8-47

**Published:** 2013-11-13

**Authors:** I Michael Leitman, Kei Suzuki, Aaron J Wengrofsky, Eyal Menashe, Michal Poplawski, Kar-Mun Woo, Charles M Geller, David Lucido, Thomas Bernik, Barbara A Zeifer, Byron Patton

**Affiliations:** 1 Departments of Surgery, Albert Einstein College of Medicine-Beth Israel Medical Center, 10 Union Square East, Suite 2M, New York, NY 10003, USA; 2 Emergency Medicine, Albert Einstein College of Medicine-Beth Israel Medical Center, New York, NY, USA; 3 Radiology, Albert Einstein College of Medicine-Beth Israel Medical Center, New York, NY, USA

**Keywords:** Acute coronary syndrome, Thoracic aorta, Aortic aneurysm, Aortic dissection

## Abstract

**Background:**

Thoracic aortic dissection (TAD) and aneurysm (TAA) are rare but catastrophic. Prompt recognition of TAD/TAA and differentiation from acute coronary syndrome (ACS) is difficult yet crucial. Earlier identification of TAA/TAD based upon routine emergency department screening is necessary.

**Methods:**

A retrospective analysis of patients that presented with acute thoracic complaints to the ED from January 2007 through June 2012 was performed. Cases of TAA/TAD were compared to an equal number of controls which consisted of patients with the diagnosis of ACS. Demographics, physical findings, EKG, and the results of laboratory and radiological imaging were compared. P-value of > 0.05 was considered statistically significant.

**Results:**

In total, 136 patients were identified with TAA/TAD, 0.36% of patients that presented with chest complaints. Compared to ACS patients, TAA/TAD group was older (68.9 vs. 63.2 years), less likely to be diabetic (13% vs 32%), less likely to complain of chest pain (47% vs 85%) and head and neck pain (4% vs 17%). The pain for the TAA/TAD group was less likely characterized as tight/heavy in nature (5% vs 37%). TAA/TAD patients were also less likely to experience shortness of breath (42% vs. 51%), palpitations (2% vs 9%) and dizziness (2% vs 13%) and had a greater incidence of focal lower extremity neurological deficits (6% vs 1%), bradycardia (15% vs. 5%) and tachypnea (53% vs. 22%). On multivariate analysis, increasing heart rate, chest pain, diabetes, head & neck pain, dizziness, and history of myocardial infarction were independent predictors of ACS.

**Conclusions:**

Increasing heart rate, chest pain, diabetes, head & neck pain, dizziness, and history of myocardial infarction can be used to differentiate acute coronary syndromes from thoracic aortic dissections/aneurysms.

## Introduction

Among the “big three” catastrophic illnesses that present with acute thoracic complaints (myocardial infarction/ischemia, thoracic aortic dissection, and pulmonary embolism) [1] differentiating between thoracic aortic aneurysms (TAA)/thoracic aortic dissections (TAD) and myocardial ischemia presents a great clinical challenge to the emergency department. The incidence of TAA and TAD are 10.4 and 2.9-3.5 cases per every 100,000 persons per year, respectively [2]. Rupture is the cause of death in approximately one-third of affected patients admitted to the hospital, although the rate of nonfatal rupture might be considerably higher [3]. Forty to 50% of patients with dissection of the proximal aorta die within 48 hours if not diagnosed and properly treated, yet, it is misdiagnosed in as many as 30% of patients [4]. On the other hand, for type A aortic dissections, those who rapidly undergo surgical treatment in experienced tertiary centers have a one year survival rate of 96% to 97.6% and a three year survival of 88.3% to 90.5%. [5]. The overall survival among recipients of thoracic endovascular aortic repair (TEVAR) stent grafts is 96%, 86%, and 69% at 1-, 3-, and 5-year follow-up, respectively [6] and 74 - 97% after open surgery [7,8]. This highlights the importance of making a prompt diagnosis of TAA/TAD.

Helical thoracic CT scanning has a reported diagnostic sensitivity of 100% and a specificity of 98% for diagnosing TAD [9]. With such accurate imaging modality, it becomes crucial to triage patients such that appropriate workup leads to prompt diagnosis in a timely manner. Making a distinction between TAD/TAA and acute coronary syndrome (ACS) is especially important as the workup of ACS is significantly different.

The early identification of patients with these rare acute aortic conditions requires astute clinical intuition. This paper examines the presentation of such patients and compares them to a cohort of patients with acute chest complaints that did not have this condition. The aim of the study was to develop a profile of patients who would be identified early in their ED course with routine screening done on most ED patients. Such information will help expedite prompt confirmatory imaging, leading to prompt and effective medical and surgical treatment.

### Patients and methods

This study was reviewed and approved by the Institutional Review Board - Human Research Committee (IRB# 106–12). A retrospective analysis of patients that presented with acute thoracic complaints to the ED from January 2007 through June 2012 was performed. Patients were identified by ED diagnosis of “aortic dissection” and “aortic aneurysm”, which were further reviewed to select only those with thoracic aortic dissection and thoracic aortic aneurysm. In addition, emergency room and inpatient hospital medical records were reviewed using ICD-9 (International Statistical Classification of Diseases and Related Health Problems) codes (441.0 – 441.9) for thoracic aortic dissection and aneurysm. In total, the study group consisted of 136 patients. Equal number of control group consisting of patients with the diagnosis of acute coronary syndrome (ACS) (primary ICD-9 414.00 thru 414.05 or secondary codes of 411.81, 411.89, 413.0, 413.1 or 413.9) were randomly chosen from the same time period and included in the study as the control group.

Demographics, physical findings, EKG, and the results of laboratory and radiological imaging were compared. Statistical analysis was performed utilizing the method of Chi-squared for categorical data and Student’s *t*-test for continuous data. A p-value of less than 0.05 was considered to be statistically significant. The data were subjected to univariate and multivariate analysis using logistic regression.

## Results

During this 5 1/2-year time period, 136 patients with initial chest complaints were found to have acute TAA only (63 patients), TAD only (49 patients) or both (24 patients) on chest CT. These 136 patients with acute thoracic aortic disease represented 0.36% of the 37,778 patients that presented with acute chest pain during the study period. The classification of the aortic pathology is listed in Table 1. The demographics and past medical history for the study group (TAA/TAD) were compared to the control group (ACS) (Table 2). When compared to the control group, study group was older (average age 69 vs. 63 years, P = 0.0034), less likely to be diabetic (13% vs. 32%, P < 0.0005), more likely to have a history of TAA/TAD (34% vs. 8%, P < 0.0001), and less likely to have a history of myocardial infarction (2% vs. 15%, P = 0.0002).

**Table 1:** Classification of pathology

	
**Thoracic aortic dissection (n = 25)**	
DeBakey I	15 (60%)
DeBakey II	5 (20%)
DeBakey III	5 (20%)
**Thoracic aortic aneurysm (n = 87)**	
Class A	33 (38%)
Class B	9 (10%)
Class C	45 (52%)
**Combined dissection and aneurysm (n = 24)**	

**Table 2:** Demographics and past medical history

**Variable**	**TAA/TAD**^**1**^	**Control**	**P-value**
**Total patients**	**136 (%)**	**136 (%)**	
Mean Age (Range)	69 (33–95)	63 (31–94)	0.0034*
Gender	69 (33–95)	63 (31–94)	0.0034*
Male	80 (59)	78 (58)	0.81
Female	56 (41)	58 (42)	0.89
Past Med History			
Diabetes	18 (13)	43 (32)	0.0005*
Previous TAA/TAD	46 (34)	11 (8)	<.0001*
Myocardial Infarction	2 (2)	20 (15)	0.0002*
Hypertension	96 (71)	88 (65)	0.37
Aortic Valve Disease	7 (5)	2 (1)	0.18
Peripheral Vascular Disease	4 (3)	2 (1)	0.68
Congestive Heart Failure	15 (11)	13 (10)	0.84
Arrhythmias	2 (1)	0 (0)	0.48
COPD^2^	10 (7)	10 (13)	0.82
Marfan’s Syndrome	3 (2)	0 (0)	0.25
Coronary Artery Disease	30 (22)	41 (30)	0.20
Atrial Fibrillation	7 (5)	7 (5)	0.78
Hyperlipidemia	4 (3)	3 (2)	1
Social History			
Smoking	46 (34)	52 (38)	0.53
Drug	18 (13)	17 (13)	1
Alcohol	33 (24)	31 (28)	0.89

^1^TAA=thoracic aortic aneurysm, TAD=thoracic aortic dissection.

^2^COPD=chronic obstructive pulmonary disease.

*Signifies statistical significance.

Presenting symptoms for the two groups are demonstrated in Table 3. Study group was less likely to complain of chest pain (47% vs. 85%, P < 0.0001) and head and neck pain (4% vs. 17%, P = 0.0007). The pain for the study group was less likely characterized as tight/heavy in nature (5% vs. 37%, P < 0.0001). While the pain was more likely to be of sudden onset (11% vs. 2%, P = 0.007), it was less likely to be increasing in severity (23% vs. 2%, P < 0.0001). Study group was also less likely to experience shortness of breath (42% vs. 51%, P = 0.01), palpitations (2% vs. 9%, P = 0.0335) and dizziness (2% vs. 13%, P = 0.0025).

**Table 3:** Pain characterization and presenting symptoms

**Variable**	**TAA/TAD**	**Control**	**P-value**
**Total patients**	**136 (%)**	**136 (%)**	
Location of Pain			
Chest	64 (47)	115 (85)	<0.0001*
Head and Neck	5 (4)	23 (17)	0.0007*
Abdominal	33 (24)	24 (18)	0.08
Extremity	15 (11)	18 (13)	0.71
Back	33 (24)	21 (15)	0.09
Type of Pain			
Pressure/Tight	4 (5)	34 (37)	<0.0001*
Squeezing	8 (10)	6 (7)	0.56
Heavy	1 (1)	7 (8)	0.11
Sharp	14 (18)	20 (22)	0.65
Migrating	27 (35)	34 (37)	0.38
No pain	22 (28)	0 (0)	<0.0001*
Duration			
Increasing	21 (23)	2 (2)	<0.0001*
Sudden	10 (11)	2 (2)	0.0165*
Persistent	7 (6)	13 (12)	0.43
Constant	36 (37)	31 (37)	0.14
Decreasing	2 (2)	4 (4)	0.84
Intermittent	21 (22)	32 (38)	0.38
Symptoms			
Shortness of Breath	48 (42)	70 (51)	0.01*
Palpitation	3 (2)	12 (9)	0.03*
Dizziness	3 (2)	17 (13)	0.0025*
Dysphagia	3 (3)	0 (0)	0.25
Chills	7 (5)	10 (7)	0.62
Fever	10 (7)	11 (8)	1
Nausea	33 (24)	42 (31)	0.28
Emesis	19 (14)	20 (15)	1
Diaphoresis	16 (12)	21 (15)	0.48
Constipation	5 (5)	1 (1)	0.22
Cough	16 (12)	21 (15)	0.48
Weakness	13 (10)	18 (13)	0.45
Altered Mental Status	9 (8)	4 (3)	0.26
Syncope	21 (15)	20 (15)	1
Wheezing	3 (3)	3 (3)	0.68

TAA = thoracic aortic aneurysm, TAD = thoracic aortic dissection.

*Signifies statistical significance.

The physical exam and radiographic findings of the two study groups are listed in Table 4. Study group had a greater incidence of focal lower extremity neurological deficits (6% vs. 1%, P = 0.04), bradycardia (15% vs. 5%, P = 0.0013) and tachypnea (53% vs. 22%, P < 0.0001). For patients that had chest x-rays (107 patients (79%) for the study group and 126 patients (96%) for the control group), widened mediastinum was associated with TAA/TAD (52% vs. 45%, P = 0.005).

**Table 4:** Physical examination and x-ray results

**Variable**	**TAA/TAD**	**Control**	**P-value**
**Total patients**	**136 (%)**	**136 (%)**	
Physical Exam			
Focal Neurological Deficit	8 (6)	1 (1)	0.04*
Heart Murmur	11 (8)	5 (4)	0.19
Aortic Regurgitation	2 (2)	0 (0)	0.48
Irregular Rhythm	5 (5)	2 (1)	0.44
Heart Rate			
Normal	92 (68)	115 (86)	0.03*
Tachycardia (>120/min)	20 (15)	12 (9)	0.19
Bradycardia (< 60/min)	24 (18)	7 (5)	0.002*
Respiratory Rate			
Normal	63 (47)	105 (78)	<0.0001*
Tachypnea (>24/min)	72 (53)	29 (22)	<0.0001*
Bradypnea (<8/min)	0 (0)	0 (0)	1
Temperature			
Normal	114 (91)	122 (95)	0.29
Hyperthermic (> 39 C)	8 (6)	4 (3)	0.35
Hypothermic (< 36 C)	3 (2)	2 (2)	1
Systolic Blood Pressure			
Normal	58 (44)	61 (46)	0.84
Hypertensive (> 180 mmHg)	64 (47)	65 (49)	0.92
Hypotensive (SBP < 80 mmHg)	11 (8)	8 (6)	0.62
Diastolic Blood Pressure			
Normal	78 (59)	90 (67)	0.22
Hypertensive (>120 mmHg)	41 (31)	34 (25)	0.37
Hypotensive (<60 mmHg)	14 (11)	10 (7)	0.50
Chest X-Ray			
Widened Mediastinum	41 (52)	26 (45)	0.005*
Tortuous Aorta	21 (27)	17 (29)	0.28
Cardiomegaly	17 (16)	15 (15)	0.49

TAA = thoracic aortic aneurysm, TAD = thoracic aortic dissection.

*Signifies statistical significance.

Laboratory results are listed in Table 5. Elevated serum blood urea nitrogen (BUN) level correlated with acute thoracic aortic disease in patients in whom this testing was obtained (70% vs. 47%, P < 0.0001). Patients with laboratory evidence of coagulopathy (elevated initial normalized ration (INR) (40% vs. 19%, P = 0.0017) or D-dimer (80% vs. 13%, P = 0.06)) were also more likely to have TAA/TAD. Serum troponin levels were higher in patients with ACS (34% vs. 18% P = 0.04).

**Table 5:** Blood test results

**Test**	**TAA/TAD (%)**	**Control (%)**	**P-value**
White Blood Cell			
Normal	93 (70)	103 (77)	0.29
Low (<3 million cells/mcL)	8 (6)	9 (7)	1
High (>12 million cells/mcL)	31 (23)	22 (16)	0.20
Hemoglobin			
Normal	80 (60)	81 (60)	1
Low (<9 grams/dL)	47 (35)	47 (35)	1
High (>15 grams/dL)	7 (5)	6 (4)	1
Hematocrit			
Normal	75 (56)	78 (58)	0.81
Low (<27%)	51 (38)	49 (37)	0.89
High (>55%)	8 (6)	7 (5)	1
Blood Urea Nitrogen			
Normal	45 (34)	69 (51)	<.0001*
Low	2 (2)	1 (1)	1
High	85 (64)	64 (48)	<.0001*
Creatinine			
Normal	71 (56)	80 (62)	0.40
Elevated (>35 mg/dL)	55 (44)	49 (38)	0.47
Lactate			
Normal	6 (60)	7 (64)	0.65
Elevated (>2.5 mEq/L)	4 (40)	4 (36)	0.89
INR			
Normal	76 (60)	75 (81)	
Elevated (>1.5)	51 (40)	18 (19)	0.0017*
D-Dimer			
Normal	1 (20)	7 (88)	0.06
Elevated (> 250 ng/ml)	4 (80)	1 (13)	0.06
Troponin			
Normal	53 (82)	80 (66)	0.04
Elevated (>0.3 ng/mL)	12 (18)	41 (34)	0.04*

TAA = thoracic aortic aneurysm, TAD = thoracic aortic dissection.

*Signifies statistical significance.

On multivariate analysis, decreasing heart rate, absence of chest pain, no history of diabetes, absence of head & neck pain, absence of dizziness, and no history of myocardial infarction were independent predictors of TAA/TAD (Table 6). There were no differences when the subgroups of patients with TAA or TAD were compared to each other (data now shown).

**Table 6:** Multivariate analysis

**Factor**	**Odd ratio**	**P-value**	**95% Confidence interval**
Heart rate	0.97	0.01	0.96 – 0.99
Chest pain	0.24	< 0.001	0.11 – 0.51
Diabetes	0.29	0.004	0.13 – 0.67
Head & neck pain	0.17	0.008	0.05 – 0.63
Dizziness	0.08	0.002	0.02 – 0.39
Myocardial infarction	0.07	0.007	0.01 – 0.48

## Discussion

An expeditious diagnosis of thoracic aortic pathology in the emergency department remains a great challenge, especially its differentiation from acute coronary syndrome (ACS) [2]. Previous studies have suggested that there are many presenting signs and symptoms for TAD/TAA but routine blood work and standard imaging have not been shown to be reliable nor reproducible [10-12]. Potential genetic markers [13] and biomarkers in rat models [14] have been proposed; however, there is a need for practical and cost effective tools that can be quickly obtained in the emergency department for the routine screening of patients with acute thoracic complaints. In the present study, we have identified factors that are typically present on admission and routine emergency medical screening.

The study group of 136 patients with thoracic aortic dissection (TAD) or aneurysms (TAA) represented a mere 0.36% of the population presenting with acute chest complaints, highlighting the difficulty in diagnosing this rare entity. It would not have been possible to employ contrast-enhanced CT scans on all such patients, especially in an emergency department that sees more than 100,000 patients per year. Pain characteristics have been shown to be unreliable in a systematic review [2,15]. The present study shows that the sudden onset in nature was more likely associated with TAA/TAD. This is in concordance with previous report by Klompas et al. [4]. On the other hand, our finding of association with increasing intensity has not been reported in other studies and may explain the evolving nature of thoracic aortic disease. On multivariate analysis, chest pain, head and neck pain, and dizziness were identified to be independently associated with ACS. These all represent easily obtainable factors in routine history taking.

As expected, past medical history for the most part was not a useful tool in differentiating TAA/TAD from ACS, as both share similar comorbidities. For example, having a history of hypertension was not a useful tool in differentiating the two disease processes. However, history of diabetes and myocardial infarction was significantly associated with ACS, both in univariate and multivariate analysis, providing another easily obtainable factor in differentiating TAA/TAD from ACS. In fact, diabetes may have a protective association against the development of aortic disease [16]. Diabetes has been shown to decrease the progression of aortic disease by direct metabolic effects by the decreased secretion of metalloproteinases from these individuals [16]. The decreased production of inflammatory cells caused by hyperglycemia in mice has also been shown to inhibit vascular smooth muscle cell death, thereby thwarting the progression of aortic disease [17]. Diabeteic patients are also more likely to develop ACS because of the proatherosclerotic and proinflammatory states associated with diabetes [18]. Our data is consistent with these findings. Diabetic patients are more likely to experience ACS than TAA/TAD.

On physical exam, we found tachypnea, bradycardia, and lower extremity neurological deficits to be associated with TAD/TAA. Of particular importance, when heart rate was analyzed as a continuous variable, increasing heart rate was independently associated with ACS.

There has been much interest in the identification of useful blood tests to make the diagnosis of TAA/TAD. Elevated plasma D-dimer levels [19-21] and plasma smooth muscle myosin heavy chain protein [22] have shown some diagnostic promise but are not routinely obtained on initial presentation. A protocol for or obtaining routine plasma D-dimer studies was not used in the present study but has been advocated by others [23]. Plasma D-dimer levels were obtained in only 13 patients in the current study (5 in study group, 8 in control), yet elevated levels showed a trend for significance. D-dimer levels may also be elevated in a large variety of other conditions, including venous thromboembolism (VTE), atrial fibrillation, congestive heart failure, disseminated intravascular coagulation and routine post-operative recovery [24]. Routine analysis on screening may therefore remains controversial. An element of coagulopathy may be a component of thoracic aortic diseases, however, as patients that presented with acute thoracic aortic disease had an elevated initialed normalization ratio (INR) compared to the ACS group. This association between elevated INR and thoracic aortic disease has been reported elsewhere [25]. Elevated BUN was associated with TAD/TAD in univariate analysis, an association that has not been reported. This may represent the physiological changes in blood flow resulting from the acute aortic injury.

It is worthwhile to note that while elevated troponin was associated with ACS in the present study, 9% of TAD/TAA cohort also demonstrated elevated serum troponin levels. Patients have been reported to have acute thoracic aortic dissection with concomitant myocardial infarction and this confusion could result in a catastrophe [26]. Thrombolytic therapy for acute myocardial infarction would be contraindicated in patients with acute thoracic aortic disease. Further confusion may be caused by EKG analysis because it has been reported that 0.1-0.2% of patients with proximal TAD will have ST-elevation myocardial infarctions occur in the setting [27]. Luo reported on six patients with clear electrocardiographic and serological evidence of acute myocardial infarction and concomitant thoracic aortic dissection, underscoring the need to be continuously vigilant for acute thoracic aortic disease, even after the diagnosis of myocardial infarction is confirmed [28].

When present, the finding of a widened mediastinum was associated with TAD/TAA, as previously reported [29]. Because a widened mediastinum is difficult to interpret on a portable x-ray, a formal standing posterior-anterior chest x-ray for patients presenting with chest pain may be necessary. CT scanning is an effective screening modality [30] but cannot be utilized for all patients with acute thoracic complaints who present to busy ED’s. Transthoracic echocardiography may also a useful imaging modality for the diagnosis of acute aortic syndromes. Some have reported it to be beneficial for screening [31] but it should not be used as the sole screening imaging technique [32].

Limitations of the study include the retrospective nature of the study design. A larger cohort of patients that presented with acute thoracic symptoms but were not found to have acute thoracic aortic dissection or aneurysm would have provided a statistically enhanced database to allow for the development of a risk prediction model. Such modeling would facilitate the use of the findings reported herein. In addition examining the missed diagnosis rate and delay in diagnosis in a prospective fashion using this model would validate the findings from this study.

Screening patients with acute chest pain in the emergency department for thoracic aortic dissection or thoracic aortic aneurysm presents a clinical challenge. In the current study, we identified increasing heart rate, presence of chest pain, head and neck pain, dizziness, diabetes, and history of myocardial infarction to be independently associated with ACS as opposed to TAA/TAD. These represent easily obtainable factors that can be used to screen patients to undergo prompt confirmatory imaging with CT of the chest.

## Competing interests

The authors declare that they have no competing interests.

## Authors’ contributions

IML conceived of the study, and participated in its design and coordination and helped to draft the manuscript. KS participated in the design of the study, performed the statistical analysis and coordination and helped to draft the manuscript. AJW participated in the design of the study, performed the statistical analysis and coordination and helped to draft the manuscript. EM participated in the design of the study and coordination and helped to draft the manuscript. MP participated in the design of the study and coordination and helped to draft the manuscript. KMW conceived of the study, and participated in its design and coordination and helped to draft the manuscript. CMG conceived of the study, and participated in its design and coordination and helped to draft the manuscript. DL participated in the design of the study and performed the statistical analysis. TB conceived of the study, and participated in its design and coordination and helped to draft the manuscript. BAZ conceived of the study, and participated in its design and coordination and helped to draft the manuscript. BP participated in the design of the study and coordination and helped to draft the manuscript. All authors read and approved the final manuscript.
